# Not All Children with Under-Control Asthma are Controlled

**DOI:** 10.2174/1874306400802010001

**Published:** 2008-02-06

**Authors:** G Ricci, A Dondi, E Calamelli, V Dell’Omo, L Pagliara, T Belotti, M Masi

**Affiliations:** Department of Paediatrics, S. Orsola-Malpighi Hospital, University of Bologna, Bologna, Italy

**Keywords:** Asthma control, bronchodilation test, childhood asthma, lung function, spirometry.

## Abstract

Subclinical lung function alterations can sometimes be discovered in asthmatic patients under clinical control. This study aimed to identify the burden of asthmatic children with subclinical airways abnormalities who may benefit from an adjustment in asthma therapy. 134 6-to-17-year-old asthmatic children were enrolled. Of them, 98 presented apparently under clinical control disease and all performed spirometry before and after bronchodilation: 17 (17.3%) had a positive bronchodilation test, in addition to significantly lower lung function indexes as compared to those with under-control asthma who had a negative bronchodilation test. These patients were randomized and re-evaluated: patients (n=8) receiving an adjustment in their therapy showed an improvement in lung function tests and quality of life indexes as compared to 7 without therapy adjustment. In conclusion, a substantial number of apparently-under-control asthmatic children show airways alterations that can be improved by adjusting their therapy, which also seems to enhance their quality of life.

## INTRODUCTION

Asthma is the most frequent chronic disease in childhood and its prevalence is increasing [1,2], but only a part of the patients reach the goals of control set out by the Global Initiative for Asthma (GINA) [3,4]. Adequate therapy may limit bronchial inflammation, thus improving the health status of the asthmatic child and preventing the disease severity from worsening [3]. The more severe asthma is in childhood, the more likely it is that the disease will persist in adulthood [5]. The main goal to be achieved in the management of the asthmatic child is to obtain a control both of the symptoms and of subtle airways alterations that can only be revealed by laboratory exams.

Lung function testing and the measurement of bronchodilation responsiveness are recommended for the diagnosis of asthma and for its monitoring over time [3]. The post-bronchodilation forced expiratory volume at the first second (FEV_1_) measures the best lung function that can be achieved by bronchodilator therapy on the day of the visit [6]; a suboptimal post-bronchodilation FEV_1_ indicates bronchial obstruction.

Several studies confirm that lung function can be altered even in patients whose disease is judged to be under control according to their symptoms referral and the use of reliever medications [4,7,9]. Collecting symptoms from the children or their parents can though be troublesome. A non-recognition of the symptoms can lead to an underestimation of the severity of asthma, consequently to inappropriate treatment, and eventually to a worsening of the disease severity.

It seems that taking into account only the symptoms as referred by the children or their parents in order to judge asthma control is insufficient [7-9]. The present study aimed to demonstrate the usefulness of performing spirometry and the bronchodilation test in asthmatic children during routine clinical visits in order to monitor their actual asthma control and severity.

## MATERIALS AND METHODOLOGY

### Patients

134 children (98 males) aged 6 to 17, who had been consecutively referred to the Allergology. Outpatient section of our clinic between November 2005 and October 2006, were included, all having a diagnosis of allergic asthma which had been established previously.

All the patients underwent a clinical examination to assess their asthma severity according to the GINA guidelines [3], and the status of asthma control on the basis of the parameters suggested by the Joint Task Force on Practice Parameters for Allergy and Immunology [10]. A child was considered to be clinically under control if, in the previous two weeks, there had been: 1) asthma symptoms twice a week or less; 2) rescue bronchodilator use twice a week or less; 3) no nighttime or early morning awakening; 4) no limitations on exercise, work, school; 5) well-controlled asthma by patient and physician assessment; 6) normal or personal best FEV_1_; 7) absence of bronchospasm at auscultation. Therapy prior to the visit was recorded.

### Lung Function Testing

All the patients performed a spirometry by means of a Multispiro SA/100 spirometer (Medical Equipment Designs, Laguna Hills, CA, USA). The best of a minimum of three recordings was chosen and a bronchodilation test was done by the administration of 200 mcg of dry salbutamol powder and repetition of the spirometry 15 minutes later. Spirometric indices were expressed as percentage of the predicted values (Polgar revisited) [11]. The result of the bronchodilation test was evaluated by examining the variation in FEV_1_ before and after bronchodilation expressed as a percentage change relative to the baseline values (deltaFEV_1_): the test was considered positive when deltaFEV_1_ was ≥12% [7,12, 13]. We chose the value of deltaFEV_1_ ≥12% instead of ≥15% to increase the sensibility of the test.

### Skin Prick Tests (SPT)

A Skin Prick Test was performed to establish possible sensitization to the main seasonal (grass pollens, *Parietaria judaica*) and perennial (*Dermatophagoides farinae*, *Dermatophagoides pteronyssinus*, *Alternaria alternatae*, cat and dog dander) allergens involved in allergic asthma (Lofarma, Milan, Italy).

### Health-Related Quality of Life (HRQL)

Patients’ HRQL was measured by administering the Pediatric Asthma Quality of Life Questionnaire (PAQLQ) [14].

### Statistical Analysis

Data were examined by means of SPSS 13.0 for Windows (SPSS/PC; SPSS Inc., Chicago, IL, USA). Student’s t-test and linear regression were used. Results were considered significant for a p value ≤0.05.

### Ethical Consideration

This research was conducted in accordance with the principles of the Declaration of Helsinki. Written informed consent was obtained from parents before the children were included in the study.

## RESULTS

According to our parameters, the asthma was under control in 98 children (73.1%), but not in the remaining 36 (26.9%). Of the 98 children with controlled disease, 17 (17.3%) had a positive bronchodilation test, meaning a deltaFEV_1_ ≥12%. This allowed us to divide our population into three different groups, one including patients with their asthma under control and with a negative bronchodilation test, the second consisting of children with controlled asthma but a positive bronchodilation test and the third including patients with uncontrolled asthma. Table **1** describes the main clinical and functional characteristics of our population. Table **2** shows the results of spirometry, bronchodilation test, Skin Prick Test and PAQLQ scores.

Firstly, differences in spirometric data between different groups in our population were examined. Results of t-test showed that FEV_1_ and the ratio between FEV_1_ and the forced vital capacity (FVC) differed significantly (p≤0.001 and p≤0.005 respectively) between patients with apparently under control asthma (with a positive or negative response to the bronchodilation test) and patients with not-controlled asthma.

Moreover, FEV_1_ and FEV_1_/FVC differed significantly (p≤0.05 and p≤0.01 respectively) between patients with asthma under control and a positive or negative response to the bronchodilation test. No significant differences were found in FVC between these groups. FEV_1_ and FEV_1_/FVC were not significantly different (p=0.180 and p=0.785 respectively) between patients with asthma under control but a positive bronchodilation test and patients with uncontrolled asthma.

Analysis of the differences in HRQL between groups showed that PAQLQ scores were similar in all children with asthma under control independently of bronchodilation test results, but a statistically significant difference was found between the scores of children with controlled asthma and a positive bronchodilation test, and children with uncontrolled disease (p≤0.01).

Examination of the allergic condition in the population did not highlight differences between the three groups regarding positive Skin Prick Test to the main allergens involved in allergic asthma (Table **3**).

Patients showing a discrepancy between the results of spirometry and asthma control were proposed to be reevaluated after a few months in order to reassess their asthma control and lung function. These patients were randomized and divided in two groups: 8 children (first group) had an adjustment in their asthma therapy, consisting of the introduction of or increase in inhaled corticosteroids (ICS), which have been shown to provide an improvement in airway responsiveness and asthma control [15]. Remaining 9 children (second group) had no adjustment in their therapy. Two children of the second group missed the visit. Table **4** shows the spirometric values and PAQLQ scores in the two subgroups of patients at the first visit. No significant differences were found between the two groups.

**Fig. (1):**
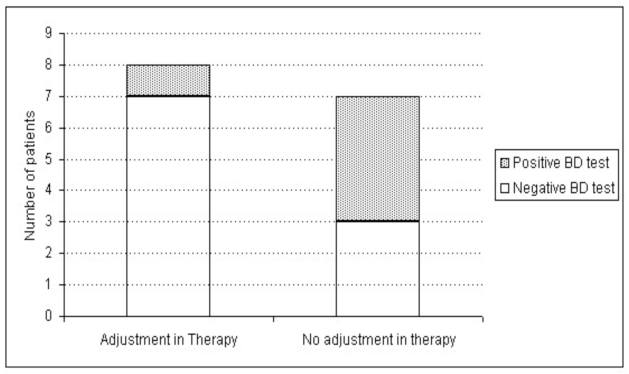
Variation in FEV_1_ before and after bronchodilation in 15 children who had previously shown a positive bronchodilation (BD) test. The BD test was considered to be positive when variation in FEV_1_ at baseline and after bronchodilation, expressed as a percentage of the baseline value, was ≥12%.

Fifteen children were re-evaluated after 1 to 4 months, 8 of whom had had their therapy adjusted and 7 had not. Control of spirometry showed that 7 (87.5%) of the 8 children with a change in their therapy had a negative response to the bronchodilation test and 1 (12.5%) had a positive response, whereas among the patients with no change in asthma therapy 3 (42.9%) had a negative bronchodilation test, and the remaining 4 (57.1%) had an increase in FEV_1_ after salbutamol ≥12% (Fig. **1**).

The same group of patients completed the PAQLQ again at the second visit. Median values showed an improvement in HRQL in those children who had been treated with ICS (6.48 at the first time, 6.72 at the second), and a worsening in children whose therapy had not been modified (6.73 and 6.39), but differences are not statistically significant. The examination of the subscales of the questionnaire indicates that 5 out of 8 children who had been treated with ICS had an improvement in the “activity limitations” domain, whereas only 2 out of 7 children who had not been treated with ICS showed this enhancement.

## DISCUSSION

The main goal in the management of the asthmatic child is to obtain control of the disease, but subclinical alterations can be occasionally revealed in the lung function of asthmatic patients under apparent clinical control. No specific guidelines could be found about the approach to this problem, even if several studies reported such discrepancies, and the fact that asthma control is poorly judged when symptoms alone are considered has already been stated [7,8,16-19].

Korhonen  in 1999 evaluated the treatment policy for asthmatic children in the area of Kuopio, Finland [8]. PEF, spirometry and bronchodilation test results were collected from 195 school-aged patients. 11% of them had a positive the bronchodilation test as the only sign of bronchial obstruction, pointing to inadequate therapy.

Our study shows a prevalence of 17.3% of positive bronchodilation tests in a group of asthmatic children who were in a symptom-free period, indicating an occult bronchial obstruction. The lung function of these children was more similar to that of children with uncontrolled asthma than to the one of those with controlled asthma and a negative bronchodilation test.

The reasons why these children are asymptomatic despite having pulmonary function impairment can be multiple. First of all, an underlying, but still subclinical, pathological process in the airways could be hypothesized, similarly to what happens in some patients with apparently outgrown disease [20-22]. Secondly, patients with asthma frequently have poor recognition or perception of their symptoms [3,22,23]. Boulet  [22] in 1994 showed that the perception of symptoms associated with airways obstruction follows a normal unimodal distribution in the population with asthma; there are some “hypoperceivers” who can be asymptomatic or have minimal symptoms even with marked reductions in expiratory flows. A poor sensibility of airways obstruction has been demonstrated when convalescing from an acute asthmatic attack, during regular follow-ups, during a stable state of asthma, and even during acute episodes [18,24-27]; some patients have difficulty in distinguishing asthmatic dyspnoea from exercise-induced physiological breathlessness [28]. Moreover, collecting the symptoms story from the parents can sometimes be misleading on the actual status because of the social and psychological impact of admitting having a child with asthma; collecting the symptoms report directly from the kids could be more accurate [29], even if children, and mainly adolescents [5], may deny symptoms.

We believe that patients with a positive response to the bronchodilation test even in the absence of symptoms should be regarded as undertreated. Our data on lung function after treatment (figure 1) show an improvement in most of the patients. On the contrary, 4 children out of 7, who were not treated after positive bronchodilation test, did not show an improvement in lung function at re-evaluation. The reason why more than 40% of children with unmodified therapy also showed an improvement in lung function can be imputed to the fact that a more stringent medical control often induces a patient’s better compliance to therapy and disease management.

The administration of the PAQLQ did not reveal any differences in HRQL between children with under-control asthma but a different response to bronchodilation. All of them, however, had a significantly better HRQL than children with uncontrolled disease. Although HRQL evaluation does not help to identify patients in need of further therapy, it is interesting to note that controlled patients with a positive bronchodilation test have some characteristics (asthma control, HRQL) in common with those well controlled and not respondent to bronchodilation, while they are more similar to uncontrolled children for other features (lung function).

However, PAQLQ re-administration in the 15 children who repeated spirometry because of the discrepancy between clinical and functional data indicates an improvement in HRQL in those who underwent ICS therapy or had an augmentation in the dosage. Their activity limitations seem to be the most concerned, because 5 children out of 8 improved in this domain of the questionnaire.

The first studies which identified such discrepancies between lung function and clinical control were conducted 20 to 30 years ago. Even if since then new drugs have been developed, asthma has been widely studied and its diagnosis and treatment have generally improved, the point of asymptomatic airways alterations is still up-to-date. Adequate treatment prevents pulmonary function from worsening: in persistent asthma, ICS suppress airway inflammation, reduce airway hyperresponsiveness, improve asthma control and prevent symptoms [3,15]. Persistent abnormal lung function in childhood, measured either at baseline or after bronchodilation, is probably associated with more severe asthma and an unfavourable prognosis [5]. The limitation of our study is that it does not include Exhaled Nitric Oxide measurements, which could add further informations on the inflammatory status and, therefore, on asthma control [30]. This device is very useful in clinical practice, but, by now, it is not still available in all pneumologic and allergologic center.

Undertreated patients are exposed to a worse progression of asthma severity and the bronchodilation test, even in the absence of Nitric Oxide measurements, seems to identify those children who are in need of a reassessment of their therapy. Moreover, our results indicate that the pharmacological treatment of an occult bronchial obstruction helps improving HRQL.

## CONCLUSION

In conclusion, we believe that it is advisable to perform spirometry in asthmatic children in order to provide adequate therapy for their actual lung condition, which can be revealed by the bronchodilation test. Establishing asthma control solely on the basis of clinical parameters could be insufficient. In the absence of Nitric Oxide measurements, spirometry is a simple examination which is normally performable in every pneumologic or allergologic outpatient clinic and allows an evaluation of the bronchial obstruction status of the lung of the asthmatic school-aged child without any further, expensive investigation.

## Figures and Tables

**Table 1.** Main Clinical and Spirometric Characteristics of 134 Children (Mean Age 10.95; 98 Males and 36 Females) Enrolled in the Study

		Under Control (n=98)		Not Under Control (n=36)	All Patients n=134
		*Negative Bronchodilation Test (n=81)*	*Positive Bronchodilation Test (n=17)*		
**Asthma severity**	***Intermittent***	*34 (42%)*	*7 (41 %)*	7 (19%)	48 (36%)
		**41 (42%)**			
	***Mild persistent***	*40 (50%)*	*7 (40%)*	19 (53%)	66 (49%)
		**47 (48%)**			
	***Moderate persistent***	*7 (9%)*	*3 (18%)*	7 (19%)	17 (13%)
		**10 (10%)**			
	***Severe persistent***	*0*	*0*	3 (8%)	3 (2%)
		**0**			
**Controller medications**	ICS*	*50 (51%)*	*8 (47%)*	17 (47%)	67 (50%)
		**42 (52%)**			
	ICS*+ LABA†	*11 (11%)*	*2 (12%)*	6 (17%)	17 (13%)
		**9 (11%)**			
	***Only reliever medications***	*37 (38%)*	*7 (41%)*	13 (36%)	50 (37%)
		**30 (37%)**			

* ICS: inhaled corticosteroids;

† LABA: long-acting β_2_agonists; bronchodilation test was considered to be positive when deltaFEV_1_≥12%; deltaFEV_1_= (FEV_1_postBD-FEV_1_at baseline) / FEV_1_ at baseline.

**Table 2 .** Results of Spirometry, Bronchodilation Test, Skin Prick Test and PAQLQ Scores

		Under Control (n=98)		Not Under Control n=36	All Patients n=134
		*Negative Bronchodilation Test (n=81)*	*Positive Bronchodilation Test (n=17)*		
**Mean FVC ± SD**		*106.% ± 13.5%*	*110%±15%*	99%±16	105%±15%
		**107%±14%**			
**Mean FEV_1 _± SD**		*99%± 12% ***§**	*92%±10% ***§**	85%±20 $	94%± 15.5%
		**97%±18% **$			
**Mean FEV_1_/FVC ± SD**		*94%±9.6% ***#**	*84%±11% ***#**	85.05%± 13 ¥	90%± 12%
		**92%±10.5% **¥			
Delta FEV_1_*≥12%		*0*	*17 (100%)*	17(47%)	34(25%)
		**17 (17%)**			
**SPT† positive results**	***Grass pollens***	*85(87%)*	*14 (82%)*	32(89%)	117(87%)
		**71 (88%)**			
	***House dust mites***	*41 (42%)*	*5(29%)*	19(53%)	60(45%)
		**36 (44%)**			
	***Cat dander***	*35 (36%)*	*4(23.5%)*	15(42%)	50(37%)
		**31 (38%)**			
**Mean PAQLQ‡ score ± SD**		*6.18±0.75*	*6.43±0.52*	5.42 ±1.18	6.02±0.93
		**6.23±0.72**			

Spirometric data are expressed as percentage of predicted. Statistics showed significant statistical differences in FEV_1_and in FEV_1_/FVC between children with apparently under control asthma and with not-controlled asthma ($: p≤0.001 and ¥: p≤0.005 respectively) and between children with under control asthma and a negative or positive response to bronchodilation (§: p≤0.05 and #: p≤0.01 respectively).

* deltaFEV1= (FEV1 post bronchodilation – FEV1 at baseline) / FEV1 at baseline;

† SPT: Skin Prick Test;

‡ PAQLQ: Pediatric Asthma Quality of Life Questionnaire.

**Table 3.** Main Allergic Characteristics of 134 Children Included in Our Study

	Grass Pollens	House Dust Mites	Cat Dander
Under control, negative bronchodilation test (n=81)	72 (88.9%)	36 (44.4%)	31 (38.3%)
Under control, positive bronchodilation test (n=17)	14 (82.4%)	5 (29.4%)	4 (23.5%)
Not under control (n=36)	32 (88.9%)	19 (52.8%)	15 (41.7%)

No differences between the three groups are statistically significant.

**Table 4.** Spirometric Values and PAQLQ Scores at the First Visit in the Two Subgroups of Patients with Positive Bronchodilation Test (n=17) who had an Adjustment in their Asthma Therapy (First Group; n= 8), Consisting of the Introduction of or Increase in Inhaled Corticosteroids (ICS), and who had No Adjustment in their Therapy (Second Group; n=9)

	Patients with Adjustment in Therapy (n=8)	Patients without Adjustment in Therapy (n=9)	All Patients with Positive Bronchodilation Test (n=17)	p Value
**Mean FVC ± SD**	107%±15%	112%±14%	110%± 15%	0.475
**Mean FEV_1 _± SD**	91%±10%	92%±10%	92% ±10%	0.840
**Mean FEV_1_/FVC ± SD**	86%±12%	82%±10%	84% ±11%	0.465
Mean Delta FEV_1_*± SD	19%±6%	16%±6%	17.5%±6%	0.320
**Mean PAQLQ‡score ± SD**	6.48±0.79	6.73±0.55	6.43±0.52	0.456

No differences between the two groups are statistically significant

* Delta FEV_1_ = (FEV_1_ post bronchodilation – FEV_1_ at vaseline) / FEV_1_ at baseline;

‡ PAQLQ: Pediatric Asthma Quality of Life Questionnaire.

## ABBREVIATIONS

BD test: = Bronchodilation test
FEV_1_: = Forced expiratory volume at the first second
FVC: = Forced vital capacity
HRQL: = Health-related quality of life
ICS: = Inhaled corticosteroids
LABA: = Long-acting β_2_agonists
PAQLQ: = Pediatric Asthma Quality of Life Questionnaire
